# Bioelectroremediation of a Real Industrial Wastewater: The Role of Electroactive Biofilm and Planktonic Cells through Enzymatic Activities

**DOI:** 10.3390/toxics12080614

**Published:** 2024-08-20

**Authors:** Laura Katherin Chaparro Díaz, Antonio Berná, Karina Boltes

**Affiliations:** 1 Departamento de Química Analítica Química Física e Ingeniería Química, Campus Científico Tecnológico, Universidad de Alcalá, Ctra. A-II km 33.6, 28871 Alcalá de Henares, Madrid, Spain; 2 IMDEA Water, Avda. Punto Com, 2, 28805 Alcalá de Henares, Madrid, Spain; antonio.berna@imdea.org

**Keywords:** microbial electrochemical technology, industrial wastewater, enzymatic activities, toxicity

## Abstract

Bioelectrochemical processes are emerging as one of the most efficient and sustainable technologies for wastewater treatment. Their application for industrial wastewater treatment is still low due to the high toxicity and difficulty of biological treatment for industrial effluents. This is especially relevant in pharmaceutical industries, where different solvents, active pharma ingredients (APIs), extreme pH, and salinity usually form a lethal cocktail for the bacterial community in bioreactors. This work evaluates the impact of the anode architecture on the detoxification performance and analyzes, for the first time, the profile of some key bioremediation enzymes (catalase and esterase) and reactive oxygen species (ROS) during the operation of microbial electrochemical cells treating real pharmaceutical wastewater. Our results show the existence of oxidative stress and loss of cell viability in planktonic cells, while the electrogenic bacteria that form the biofilm maintain their biochemical machinery intact, as observed in the bioelectrochemical response. Monitorization of electrical current flowing in the bioelectrochemical system showed how electroactive biofilm, after a short adaptation period, started to degrade the pharma effluent. The electroactive biofilms are responsible for the detoxification of this type of industrial wastewater.

## 1. Introduction

In recent years, the global demand for pharmaceuticals and personal care products (PPCP) has shown a continuous increment due to population growth and aging [1]. The consumption trend has surged since 2020, caused by the global pandemic situation [2]. The pharmaceutical sector continues to increase the manufacturing of active pharmaceutical ingredients (APIs) that reach the market for consumption. The growing manufacturing of APIs is associated with greater water consumption and the generation of industrial liquid effluents. These pharmaceutical wastewaters have inherent complexity and variability, are highly toxic for aquatic organisms, have high chemical oxygen demand (COD), containing a wide variety of organic and inorganic constituents [3]. Numerous components of real pharmaceutical wastewaters are characterized by their low biodegradability and high persistence in the environment, whose presence in low concentration levels (µg/L-ng/L) is not routinely monitored and can cause ecological disruption and may trigger unwanted ecological effects by exerting stress on the organisms, falling into the category of so-called emerging pollutants [4,5].

There are multiple studies reporting the presence of PPCP, in wastewater, surface water, and groundwater [6,7,8]. Aquatic ecosystems are the most sensitive to the wastewater discharged because they are the primary receptors of the effluents of wastewater treatment plants. Previous studies have investigated the occurrence of pharmaceuticals in the effluent and influent of wastewater treatment plants and found that the removal of these micropollutants is incomplete because the treatment plants are primarily designed for the biodegradable removal of organic matter and not for the removal of these organic micropollutants [9,10,11]. In addition, it must be taken in mind that most of the components of pharma sector effluents are bioactive substances because they have been specially designed to modify biochemical functions in humans and animals. For this reason, wastewater generated in pharmaceutical factories can also affect the enzymatic activities and metabolic mechanisms of living organisms [12].

Therefore, the treatment of this type of wastewater requires technology capable of efficiently removing the active pharmaceutical ingredients and other substances present in the effluent. Several studies have been published on the use of physicochemical treatments to remove these organic micropollutants in pharmaceutical effluents. In this line, it was proposed adsorption [13], electrocoagulation [14], membrane filtration [15], ozonation [16], and other advanced oxidation techniques [17]. All of these physicochemical processes present different weaknesses, especially derived from their high energetic operational cost [18]. In addition, in the case of adsorption and membrane filtration, this treatment is an incomplete solution since the contamination is concentrated and transferred to another phase or stream [19,20].

In the field of biological treatment, an important drawback is their low efficiency due to the toxicity of effluents for the organism that carried out pollutant metabolization, the lack of specific microorganisms with the capacity to eliminate the different compounds, and the low availability of electron donors and acceptors that support microbial metabolisms [21]. Microbial electrochemical technologies (MET) emerged as the most promising biological process to carry out wastewater decontamination [22,23,24]. MET performs biologically catalyzed processes capable of converting chemical energy from organic matter directly into electricity or other by-products through the mediation of pure/mixed cultures of electroactive bacteria (EAB) [25,26] with the interaction of electroconductive materials [27,28].

There are different MET-based system configurations previously tested for the removal of pharmaceuticals, horizontal flow bioelectrochemical filters [29], and microbial electrochemical fluidized bed reactors (ME-FBR) [30]. A lot of work has been also published on the removal of pharmaceuticals using mono and dual chambers Microbial fuel cells (MFC) [31,32,33] and microbial electrolysis cells (MEC) [1,34,35,36]. Published works can also be found reporting a combination of MFC plus constructed wetlands [37,38]. Most articles studied synthetic wastewater and urban wastewater, and studies conducted using real pharmaceutical wastewater are scarce. However, authors agree that bioelectrochemical technology considerably improves the removal yield of organic matter and organic micropollutants as well as their associated toxicity.

In general, all these previous works focus on electrode configurations and operational conditions to reach the maximum removal of pollutants. The current generation potential and microbial ecology on the electrode surface is another highly studied aspect of the development of MET technology. Moreover, there are limited studies in the wastewater treatment field at a mechanistic level, in which the role of enzymes involved in the biodegradation and detoxification process are investigated [39]. Enzymes have been shown to play an important role in the degradation and biotransformation of pharmaceuticals in anaerobic sludge [40,41,42] due to enzymes ultimately catalyzing the metabolic or cometabolic transformation of micropollutants [43].

Previous work confirmed alterations in the enzymatic activity, such as lower catalase activity and the increment of reactive oxygen species causing oxidative stress in anaerobic and aerobic bioreactors due to the presence of pharmaceuticals [44,45,46]. Some works have been recently published in which it was evaluated enzymatic activities in the MET systems. The constructed wetland with MFC, integrated or polarized, were operated using synthetic wastewater [47,48] or urban wastewater [49]. Enzymatic activities like dehydrogenase, catalase, and hydrolase were determined under different operational conditions. Authors agree that bioelectrochemical systems showed the best enzymatic activities compared to the non-bioelectrochemical controls. Additionally, one article [50] studied the induction of antibiotic resistance genes (ARGs) by the antibiotic sulfamethoxazole (SMX) in MFCs treating synthetic wastewater. Here, the authors evaluated enzymatic activities, showing that *Geobacter* (the most representative electroactive bacteria) generates oxidative enzymes to facilitate the co-metabolism of the antibiotic and acetate (their carbon source).

Until now, no work has been published that analyzes alterations in enzymatic activities in bioelectrochemical systems treating highly contaminated and toxic effluents. Previous works studied synthetic effluents similar to urban wastewater (low organic content), and the ecotoxicity was not determined. Moreover, in the previous articles, in which some enzymatic activities were determined, no discrimination has been made between electroactive biofilm-forming cells and planktonic cells. Therefore, there is a knowledge gap regarding the enzymatic response and alterations caused by toxic compounds on active microorganisms in bioelectrochemical reactors. This work aims to contribute to this knowledge by specifically studying the level of oxidative stress in planktonic cells and electroactive bacteria colonizing the anode of the bioelectrochemical devices used, measuring reactive oxygen species (ROS), catalase activity, and esterase activity.

Oxidative stress in bacterial systems, in the presence of toxic compounds, is a complex process involving the generation of reactive oxygen species, subsequent damage to crucial cellular components, and a series of cellular responses to mitigate this damage [51]. The ability of bacteria to handle oxidative stress is crucial for their survival in hostile environments such as industrial wastewater treatment reactors, where cell viability is continually compromised by continuous exposure to numerous pollutants. Under these toxic stress conditions, bacteria respond in a non-specific manner, generally by generating reactive oxygen species (ROS) and increasing the activity level of certain enzymes directly related to oxidative stress (e.g., catalase) to counteract oxidative stress. However, high levels of ROS lead to cell death. This loss of cell viability can be assessed by esterase activity, which allows the assessment of cell integrity through the occurrence of intracellular reactions with the formation of fluorescent products [52]. This non-specific response to the presence of toxicants has not been previously studied in bioelectrochemical systems, where it is necessary to understand the mechanism of detoxification and the way in which the mixed and complex population of microorganisms that forms the biomass of this new generation of bioreactors is organized, self-protected and acts.

This work aims to analyze for the first time the alterations in key enzyme activities in response to toxic substances in a real, complex, highly toxic industrial wastewater and whether the profile of enzyme activities differs between electroactive biofilm-forming cells and the planktonic cells. Furthermore, this work aims to explore if the anode architecture could impact the biological activity, to the enzymatic level, of the biomass in the system, discriminating the effect for immobilized cells and those free in the liquid medium of the reactor.

## 2. Materials and Methods

### 2.1. Reactor Design and Operation

Two microbial electrolysis cells (MEC) were operated in parallel under continuous feeding at a hydraulic retention time of 24 h. Reactors have a double chamber (size 6 cm × 6 cm × 2 cm; 300 mL volume), separated by an anion exchange membrane FAS-PET-77 (Fumatech, Bietigheim-Bissingen, Germany), with 75 µm of thickness. Electrodes of Ag/AgCl 3M (HANNA) were used as reference. The difference between these reactors was the materials of their working electrodes (anode). One reactor was constructed with a graphite rod (5 cm × 1 cm, surface geometrical area 16.5 cm^2^) (MEC-R) in the anode. The second reactor used carbon felt (2.5 cm × 2.5 cm × 0.5 cm, surface geometrical area 17.5 cm^2^) (MEC-F). In addition, carbon felt was also used in the cathode of both systems.

The choice of materials was intended to address the well-known effect of electrode material on the bioelectrochemical activity exhibited by electroactive biofilms either in MFCs or MECs. This effect has attracted the attention of many researchers, not only in the past [53,54,55]. Carbon felt and graphite rods are two conventional materials used for comparison in all those studies, where the main differences have been identified as the huge surface area of carbon felt due to porosity or the different affinity for the growth of electroactive biofilms in these materials [53,54,55]. For comparison purposes, electrodes of these two different materials but with the same geometrical area were prepared in order to check whether the carbon felt porosity in the nanoscale range would affect the enzymatic activity of the electroactive biofilms developed on these electrodes [56].

The geometrical area was used as a reference, but the real active surface area was not directly determined. Implicitly, the electrochemical response of each kind of electrode is influenced by the different electrochemical active areas interacting with microorganisms, resulting in the growth and development of different electroactive biofilms. It is clearly observed in the different order of magnitude in the electrical current recorded in each case.

The graphite employed as electrode material in this work was the same (provided by Metfilter, Madrid, Spain) reported and characterized in this work by Prado et al. [57], where it was determined that this material has neither meso nor microporosity, so the real surface area meanly matches the geometrical area.

In the case of carbon felt, it is RVG4000 (provided by Mersen, Barcelona, Spain), and the specific surface area value informed by the manufacturer is in the range of classical carbon felts employed in electrochemistry, 0.2–0.4 m^2^/g [58]. All the statements and discussions incorporated in this work are based on this information about the characteristics of the chosen electrode materials.

The wastewater used in this study was taken from a pharmaceutical company located in Alcalá de Henares (Madrid, Spain). This industry treats its liquid effluent by physicochemical pre-treatment, followed by an aerobic biological oxidation process with pure oxygen supply, and finally by membrane filtration and subsequent discharge to a municipal wastewater collector that takes this treated effluent to one of the city’s wastewater treatment plants. The real effluent used in this work does not come from an anaerobic biological reactor; on the contrary, it is treated in an aerobic reactor that supplies pure air. The wastewater has been taken from the first stage of biological treatment, which consists of a process with a very short contact time for the adsorption of contaminants. In the real plant, there is a second phase of biological treatment with a longer residence time for biological oxidation, but the wastewater used does not correspond to the water output from this second stage but to the first, so the degradation is incomplete and retains high ecotoxicity. In view of the above, the indigenous microorganisms of the industrial treatment system were not anaerobic, and a prior inoculation period was required to develop active biomass to carry out biological treatment in MEC or conventional reactors.

The anodic chambers of the MEC systems and the control non-electrochemical reactor were inoculated with anaerobic granular sludge from a wastewater treatment plant (Guadalajara, Spain) plus an active culture of *Geobater sulfurreducens* and operated in batch mode for 24 days for acclimation and biomass growth. During this stage, mineral nutrients and sodium acetate were used, similar to the previous work of our research group [29,30]. After that, all systems were continuously fed with real wastewater taken from a pharmaceutical industry near to Alcalá de Henares (Spain) without additional nutrients, using a peristaltic pump (Heidolph, PD 5006). In this continuous feeding, a hydraulic retention time of 24 h was used. The cathodic chambers were re-circulated with distilled water. In addition, biological control systems were operated in parallel without polarization. The control reactors had the same construction as the MEC reactors (without electrodes), were inoculated with the same sludge, and fed with the same pharmaceutical wastewater. The experimental working conditions used in this work are shown in Table 1.

**Scheme 1:**
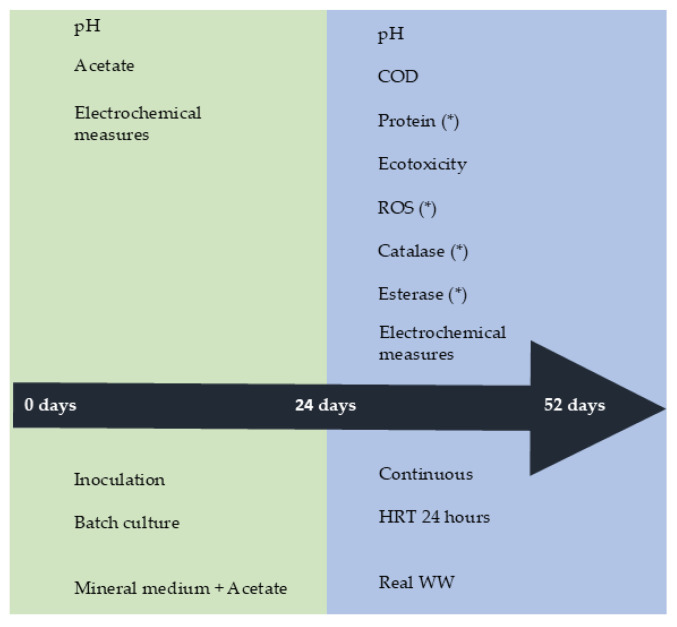
The experimental procedure is used for inoculation, continuous operation, and measurements of bioelectrochemical and control systems. * Denotes parameters determined at the end of experiments.

All bioreactors were operated in continuous (HRT 24 h) for 28 days. The following parameters were monitored daily in the experiment: pH, COD, ecotoxicity, protein, reactive oxygen species (ROS), enzymatic activities, and current. The toxicological profile of the influent and effluents taken from the bioreactors was obtained using two different organisms, the marine bacteria *Vibrio fischeri* and an aquatic plant *Spirodela polyrhiza*. Similarly, ROS and enzymatic activities, esterase, and catalase, were determined in the same effluents. Enzymatic activities, ROS, and protein in anodes were determined at the end of the continuous operation. The operational phases and measurements are shown in Scheme 1.

### 2.2. Chemical Analysis

pH was measured with a CRISON pH meter. COD was determined using a HACH commercial kit (Berlin, Germany) by adding 3 mL of sample diluted 1:2 with distilled water. Samples for COD were digested during 2 h at 148 °C in a thermoreactor TR 420 (MERK) and then analyzed in the spectrophotometer NOVA 60A.

### 2.3. Enzymatic Activity

Several tests were carried out to determine the biochemical activity of the anodic electroactive biofilm and the planktonic cells in bioreactors. The accumulation of ROS, esterase activity, and catalase activity was determined according to the methodology reported in [44,45]. The results of all activities were corrected by the protein content of samples measured by a QUBIT protein assay kit (Thermo Fisher Scientific, Waltham, MA, USA).

At the end of the experiments, cells of the working electrode were demobilized by vortex for 10 min in 30 mL of phosphate-buffered saline (PBS). Samples were centrifuged (10,000 rpm for 5 min) and washed with PBS. This fraction was used to determine enzymatic activities in whole cells collected from the electroactive biofilms. The enzymatic activity of planktonic cells of reactors was directly measured in the effluents.

To determine ROS and catalase activity, 10 mL of cell suspension were sonicated for 5 min at 1 W/mL using a Sonics-VibraCell ultrasonic cell disintegrator (BioBlock Scientific, Illkirch Cedex, France). The sonicated suspensions were centrifuged (10,000 rpm, 2 min, 4 °C) and filtered by Nylon membrane 0.2 µm pore size, collecting the filtrates for the measurements.

#### 2.3.1. Esterase Activity

Fluorescein diacetate working solution was prepared by dissolving 20 mg of fluorescein diacetate (FDA) in 10 mL of dimethyl sulfoxide (DMSO). The assays were carried out in 96-well microplates (Black F96, Nunc; Fisher), adding 195 µL of the bacterial suspensions in PBS or the effluent of reactors and 5 µL of fluorescein diacetate working solution. The fluorescence emission was measured at an excitation wavelength of 485 nm and an emission wavelength of 528 nm for 30 min, using a Fluoroskan Ascent FL microplate reader (Thermo Scientific™).

#### 2.3.2. Catalase

The catalase activity was determined in the supernatant of the sonicated samples, according to the procedure explained in Section 2.4. Here, 1.5 mL of sample plus 0.5 mL of 0.003% v/v H_2_O_2_ were incubated under constant stirring for 5 min. The oxygen production rate was measured by the OXYGRAPH SYSTEM (Hansatech, Petney, UK).

#### 2.3.3. Reactive Oxygen Species (ROS)

A stock solution of DCFH-DA was prepared by adding 200 µL of 5 mg/mL of DCH-DA in DMSO and 50 mL of phosphate buffer pH 7.4. This solution was stored at −20 °C until use. The test was carried out into a 96-well microplate (Black F96, Nunc; Fisher). 150 µL of samples (prepared according to the procedure in Section 2.3) plus 50 µL of DCFH-DA stock solution were incubated for 30 min at 37 °C. The fluorescence emission was monitored for 30 min using a Fluoroskan Ascent Fluorometer (Thermo Scientific™), using an emission wavelength of 485 nm and an excitation wavelength of 530 nm.

### 2.4. Ecotoxicity Evaluation

Ecotoxicity tests were carried out using two different biosensors, the duckweed *Spirodela polyrhiza* and the marine bacterium *Vibrio fisheri*. Tests were performed with the influent and effluent of bioreactors.

#### 2.4.1. Ecotoxicity Using Aquatic Plant

The test was carried out following the International Standard ISO 20227:2017, with some modifications. Turions of *Spirodela polyrhiza* was purchased from MicroBioTests, Gent, Belgium. According to the supplier, turions were incubated for 3 days at 25 °C in Steinberg medium for germination. Germinated plants were transferred to transparent plates. Each cell was filled with 1 plant and 1 mL of reactor influent or effluent. Reference tests were also prepared, incubating 1 plant into 1 mL of Steinberg medium. Each condition was replicated 6 times. Plates were incubated at 25 °C with 6000 lux irradiations in a growth chamber (IBERCEX, Arganda del Rey, Spain) for 72 h.

Images of plants were obtained daily at 0, 24, 48, and 72 h of exposure time. The size of the first frond, determined by Image J software (version 1.54f), was used for the evaluation of the growth inhibition percentage by comparison with the reference test without contaminants. The calculation was Carried out using Excel and the statistical by RStudio software (AGPL v3).

#### 2.4.2. Ecotoxicity Using Marine Bacteria

The toxicological level of real wastewater along treatment was also determined following the procedure of ISO 11348-3,2007, with some changes. Marine bacterium from BioTox™1243-1000 WaterTox™ Standard kit was purchased from MicroBioTests (Belgium). To determine the inhibition of the bioluminescence emitted by this bacterium in contact with reactor effluents, samples were osmotically adjusted with 2% NaCl (Suprapur). Lyophilized bacteria were reactivated and temperature-conditioned, according to the supplier’s instructions. Tests were conducted in white 96-well microplates. Each condition was replicated 8 times, and light emission was continuously monitored by Fluoroskan Ascent luminometer (Thermo Scientific™) at 1 min intervals. Finally, the data of luminesce emission in samples were compared with light emission measured in reference control (*V. fischeri* in the standard medium) to determine the bioluminescence inhibition in percentage with respect to the reference test. The calculation was carried out using Excel and the statistics by RStudio software.

### 2.5. Electrochemical Measures

MEC were continuously polarized using potentiostat NEV 2 (Nanoelectra, Madrid, Spain) to 200 mV. In order to evaluate the electrical perform of the anodic biofilm, chronoamperometries were carried out, collecting data through the KEITHLEY instruments system (Model 2700, Cleveland, OH, USA) every 60 s.

## 3. Results and Discussion

### 3.1. Characterization of Real Effluent

Characteristics of the real pharmaceutical wastewater used in this work are shown in Table 2. Here, it can be observed that there is not a high level of COD in comparison with other types of industry wastewater, but wide variations in the characteristics of the wastewater effluents in pharma sector is recognized [59,60]. The composition of effluents generated in pharmaceutical factories varied depending on the use of solvents for synthesis reactions and product separations, catalysts, reactants, sugars, starches, and nutrients from fermentation, and residues of all the APIs produced. The industrial wastewater used in this work has a high level of pH value, which is frequently observed in effluents from the pharmaceutical industries [61].

It is practically impossible to determine all the components of complex effluents. Therefore, and considering the reinforcement of water quality regulations for discharges as well as the increased level of protection of aquatic ecosystems worldwide, the ecotoxicity of these complex effluents is a good indicator of the damage they could cause to representative organisms in the receiving watercourses. Even though the pharmaceutical industry has a large economic activity worldwide, there are not many studies that report the ecotoxicity values of actual effluents. In this regard, the work of Maselli et al., 2015 [62] is one of the few where ecotoxicity on different biosensors was systematically studied. The authors evaluated the toxicity of real effluents before and after treatment, observing that although there is the removal of several contamination parameters, such as organic matter, the toxicity of the effluent was not significantly reduced. Likewise, the authors reported that the organisms most sensitive to these real effluents studied were microinvertebrate > green algae > marine bacteria > plant.

As shown in Table 2, 100% inhibition was determined for both ecotoxicity biosensors for the aquatic plant *S. polyrhiza* and the marine bacteria *V. fisheri*. In addition to the toxicity of the real pharmaceutical wastewater, it was highly toxic to aquatic ecosystems, probably due to the complex mixture of organic and inorganic compounds in this effluent. These values are in accordance with reported data in [62], in which the luminesce of the marine bacteria *V. fischeri* was completely inhibited by all the untreated real effluents assayed. For the plant *Lactuca sativa*, authors reported variable toxicity of untreated wastewater corresponding to 62–88% of growth inhibition. Ecotoxicity of the pharma wastewater previously reported on plants resulted in lower than the value obtained in this work, but this difference could be due to organisms used as toxicity biosensors. In phytotoxicity studies, *L. sativa* and *S. polyrhiza* are commonly used plants. However, there are differences between the two plant species that are determinants in the observed negative effects. *L. sativa* was grown in soil, and *S. polyrhiza* was grown in a water medium. For the first one, the germination rate and root length are the parameters determined but for the second organism, the number of fronds and biomass growth is the endpoint usually evaluated.

### 3.2. Profile of Enzymatic Activities and Oxidative Stress Level

**Figure 1:**
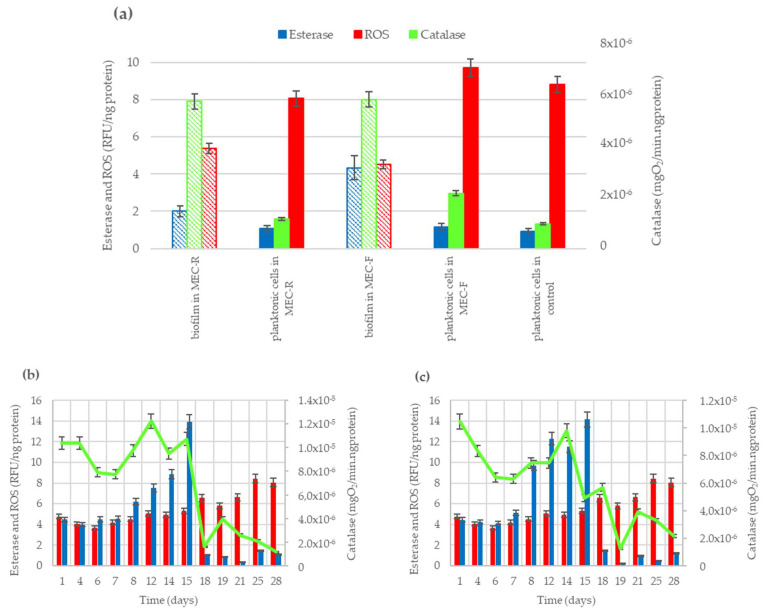
Enzymatic activities (esterase and catalase) and oxidative stress level (ROS) for fixed and free cells in bioreactors. (**a**) Values at day 28 in anodic biofilms (grid pattern bars) and planktonic cells (solid color bars); (**b**) time profile of enzymatic activity for planktonic cells in the MEC system with anode of carbon rod; (**c**) time profile of enzymatic activity for planktonic cells in the MEC system with an anode of carbon felt.

Figure 1 illustrates the measured values of esterase and catalase activities at the final experimental time (28 days of continuous feeding), as well as the oxidative stress level (ROS), in both biofilm-forming cells and planktonic cells within the bioreactors. Figure 1a displays the enzymatic parameters obtained for cells attached to the anode (rod and felt), which characterize the EAB biofilm at the enzymatic level and assess its oxidative stress in terms of ROS. This panel also includes the esterase and catalase activities and the oxidative stress levels measured in planktonic cells collected from the effluent of both bioreactors. Additionally, for comparison, the values of the same parameters for planktonic cells obtained in the control reactor are presented.

Attending to the esterase and catalase activities determined for planktonic cells at the end of the experiments, in Figure 1a it can be observed that the obtained values for planktonic cells were always lower than the values determined in biofilm-forming cells. The final esterase activity value in the biofilm of the MEC-R system resulted in double the activity of their planktonic cells. In the same way, the esterase activity in the biofilm of the MEC-F system was four times greater than the activity of their planktonic cells. Similarly, catalase activity in biofilm was five-fold higher than the obtained for planktonic cells in MEC-R and was three-fold higher in MEC-F systems. Comparing the ROS levels measured, it can be observed that planktonic cells are exposed to 1.5 times higher stress level than biofilm-forming cells in MEC-R and 2.1 times higher stress level than biofilm in MEC-F system. In reference [50], it was reported that EAB responds to the presence of the antibiotic sulfamethoxazole, generating catalase enzymes to counteract ROS levels and help the biofilm resist the action of this pollutant. In this previous work, the enzymatic activities and ROS levels of planktonic cells were not determined.

The protein concentration was determined in all samples collected during the operation time of the electrochemical systems (MEC-R and MEC-F) and the control reactor. These samples allowed us to know the protein concentration related to the planktonic biomass. Initially, both MEC systems had similar protein concentration values, near above 4000 ng/mL, but these values evolved differently. The final protein concentration values were lower in the effluent of MEC-F compared to MEC-R. The protein concentration in the effluent of the MEC-F reactor has progressively decreased over the operation time to half the initial value. However, the protein concentration has increased more than 75% for the MEC-R reactor. A gradual decrease in protein concentration has also been observed in the control reactor, with a faster decline than in the MEC systems, resulting in a final concentration 50% lower than the initial value.

The measurement of the protein concentration of the biofilm cannot be carried out during the operation since it involves the destruction of the biofilm (removal of EAB cells). However, it was evaluated at the end of the experiment. In this case, the protein content in the sample of the MEC-R anode is 24% lower than in the MEC-F. Changes in protein concentration during a biological process could be indicative of variations in biomass concentration, which is a consequence of stress situations. In this work, these differences in protein concentration levels have been considered to correct the enzymatic activities and ROS levels measured over time in all reactors.

Regarding the results obtained in biofilms (pattern bars in Figure 1a), the catalase activity values determined in cell demobilized from anodes resulted similarly in bioreactors using carbon rod (MEC-R) or carbon felt (MEC-F) as the anodic material, despite the differences in biomass content in the biofilm formed on rod and felt anodes, estimated by total protein test. Significative difference was obtained in esterase activity, resulting in 2.2-fold higher content of cells attached to the felt anode compared to the value measured in cells attached to the carbon rod anode. Similarly, biofilm formed on the carbon rod is exposed to a ROS level 20% higher than the biofilm attached to carbon felt.

On the other side, Figure 1b,c shows the time profile of enzymatic activities of planktonic cells in MER-R and MEC-F systems, respectively. Here, it can observe similar patterns for catalase activity in planktonic cells of both MEC reactors. The green lines denote a period of higher catalase activity until 15 days of operation. After that, catalase activity begins to decline, becoming more rapid in the MEC-R system. The continuous loss of catalase activity also occurs in the MEC-F system, but more slowly. Comparing the values of catalase activities in Figure 1b,c, it can be observed that catalase declined over 2.2 RFU/ng-protein.days in the MEC-F system vs. 3.3 RFU/mg-protein days in the MEC-R system.

Likewise, in Figure 1b,c, the loss of catalase activity of the planktonic cells determined an increment of the oxidant species concentrations in both reactors (red bars) and a decrease in esterase activity (blue bars). The ROS levels measured in both the MEC reactors also have similar profiles, showing a stable value until 15 days, from which it increased by 60% regardless of the anode architecture used in the reactors. The esterase activity, on the other hand, has been gradually increasing in both systems until day 15, when a significant drop can be observed, resulting in very low levels of cell viability for the planktonic cells.

The trend of enzymatic activities and ROS levels obtained in the MEC reactors show the loss of cellular viability of planktonic cells. The planktonic cells in both reactors were over the pressure of high levels of oxidative stress from day 18 until the end of the experiment.

Wang et al., 2024 [48] studied the removal efficiency of COD and nitrogen for different configurations of constructed wetlands. The authors also determined biological activity in systems in terms of microbial diversity and enzymatic activity. Here, synthetic wastewater with very low COD content was used, and no toxicity was measured. The configuration of these reactors differs greatly from the bioelectrochemical systems used in the present work. The constructed wetlands studied in [48] are granular-filled reactors made of conductive materials (such as activated carbon) and non-conductive adsorbent materials (such as sand or gravel), and they were operated in the MFC mode or in an open circuit. In this work, the bioreactors are dual-chamber devices operated in the MEC mode, as explained in Section 2.1. In the work of Wang et al., 2024 [48] it was reported higher enzymatic activity values in the anodic material (made of activated carbon) of the electrochemical systems and an increment of catalase activity by 8.72% compared to activated carbon used as a filling mat (no as anode in the MFC mode). Regarding the values reported for enzymatic activities, they are difficult to compare to the ones obtained in our work due to the different ways in which these values are obtained. The authors determined the number of enzymes per µmol of substrate consumed in 1 min and reported by a gram of activated carbon taken for analysis, and the values for planktonic cells were not measured. In this work, the enzymatic activities were reported by nanograms of total protein collected from anodes or free cells, so the comparison is direct between both types of biomass in electrochemical systems. Our results are, however, in agreement with the previous work since the enzyme activities (catalase and esterase) are higher in the anodes of both bioreactor configurations studied.

Likewise, in reference [49], where polarized and non-polarized constructed wetlands were also studied, the authors reported that the enzymatic activity, measured by FDA hydrolysis, was higher in polarized systems compared to those operating in the MFC mode and conventional non-bioelectroactive system. This previous work is also in agreement with the results obtained in the present work, where the anodic biofilm presented the highest capacity to hydrolyze FDA and reinforces the idea that bioreactor polarization stimulates the biological activity of the cells.

### 3.3. Detoxification and COD Removal

**Figure 2:**
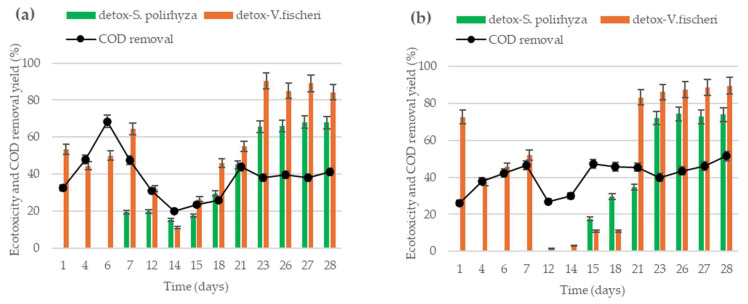
The time evolution of COD removal yield and detoxification yield determined using the aquatic plant *S. polirhyza* and the marine bacteria *V. fischeri*: (**a**) values obtained in the effluent of MEC-R system, using carbon rod as anode; (**b**) values obtained in the effluent of MEC-F reactor, using carbon felt as the anode.

Figure 2 shows the time evolution of COD removal yield and ecotoxicity removal yield, determined with two toxicological sensors, for both systems along the complete experimental operation.

Figure 2a presents the results for MEC-R systems, in which fluctuations can be observed in the COD removal yield, including a maximum value of 68.5% at day 6 of operation. After that point, COD removal decreased to a minimum value of 20% on day 14 of the operation, from which it began to increase again, reaching a stable value close to 40% removal. The same trend was observed for detoxification yield obtained with the marine bacteria as biosensors. As can be observed in Figure 2a, the detoxification yield measured with *V. fischeri* showed a minimum value of 11% on day 14 of the operation, reaching 84% at the end of the experiment. In addition, ecotoxicity was determined using the aquatic plant S. polyrhiza, which resulted in non-detoxification before day 7 of operation. After this point, the toxicity effect of effluent on the aquatic plant increased gradually, reaching a stable yield near to 68%.

Figure 2b shows results obtained in the MEC-F reactor. COD removal yield presented variation, showing a maximum value of 47% at day 7 of operation, from which decay to a minimum value of 27% reached at day 12 of the operation. After that, COD removal yield increased gradually, reaching 52% at the final time. Attending to the ecotoxicity results, it can be observed that detoxification yields determined with marine bacteria follow the same profile that COD removal. At the initial time of operation, detoxification was higher, but it was fallen near to 1% at date 12 of continuous operation. After that, detoxification increased to reach stable values of around 89%. In the same way, detoxification measured with the aquatic plant as biosensors denotes a similar profile to the marine bacteria. Here, phytotoxicity of effluents started to decrease on day 15 of operation, reaching a maximum and stable value of 74%.

Continuous exposure of microorganisms in the treatment systems to toxic substances in the feed leads to alterations that can even change the composition of the biomass. In conventional treatment systems, the presence of heavy metals has been reported to alter biological activity, reducing C and N removal performance [63], but the bacterial population can develop adaptive strategies and restore removal levels after long periods of operation (around 30 days for aerobic suspended biomass). Modifications of the biological treatment capacity and bacterial population structure have also been reported in reactors with granular biomass, which eventually developed adaptive responses to cope with the toxicity of the feeding [64].

This variation is consistent with the changes in detoxification performance measured over time, which presents a minimum coinciding with the minimum COD removal capacity. Likewise, despite using two different toxicity biosensors with different sensitivities to toxicants, the detoxification curves show similar trends, and the minimum value is located precisely at the minimum of COD removal. This result reinforces the idea of a combined toxic effect of metabolites and the parents’ pollutants of the wastewater.

Considering the results shown in Figure 2a,b it can be observed that both reactors show similar profiles. Removal yields (COD and toxicity) presented minimum values between days 12 and 14 of operation. The improvement of the organic matter removal and toxicity elimination performances are faster in the MEC-F reactor and are still growing at the end of the experiments (28 days).

### 3.4. Electrochemical and COD Analysis

Bioelectrochemical systems provide a unique tool for monitoring microbial activity: the electrical current flowing through the system. This parameter can be measured through chronoamperometry in the MEC configuration [27].

**Figure 3:**
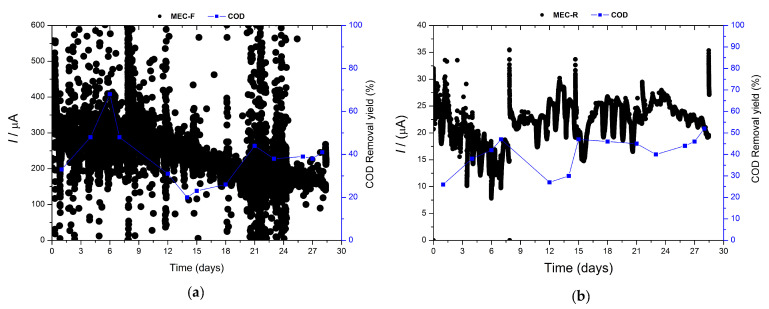
Chronoamperometry and efficiency removal of COD and both MEC: (**a**) MEC-F, (**b**) MEC-F.

In Figure 3, chronoamperometry assays are displayed for both electrodes, felt, and carbon rods. The graph corresponding to MEC-F exhibits a major production of current compared to MEC-R because the electrode area of the felt was bigger than the rod. Nevertheless, the electrical current had been stable during the time in both cells. On the other hand, as shown in Figure 3, the chemical oxygen demand values show a similar pattern to the one exhibited by toxicity, Figure 2. At the beginning, the starting electrical current values were very high but showed a decreasing trend during the experiment. This trend is more obvious in the case of MEC-R rather than MECF. After some time of operation, the electrical current flowing in the system attains a stable value for the last two weeks of the experiment in both cases.

In previous work by our research group [65], it was shown that a bioelectrochemical-constructed wetland, operated for 525 days with real urban wastewater, gradually increased COD removal performance, showing downward and upward variations in COD removal when subjected to increases in organic loading rate, but a stable electrical current of 100 mA was maintained during the long-time operation. This behavior was identified as evidence of no electron acceptor limitation in the system. Therefore, this fact was assigned to the contribution of alternative bioremediation processes like fermentation where final electron acceptors alternative to the electrode were being used. Typically, the contribution of processes using final electron acceptors different from the electrode becomes significant at high organic load values. Furthermore, the absence of variations in the value of the recorded electrical current when the organic load removal rate decreases can be assigned to the resilience of the electroactive biofilm in comparison with the resilience exhibited by planktonic cells.

Based on these previous results, the variations observed in COD removal along the operation of the MEC systems with both configurations of anodes used are considered normal and can be correlated with adaptive processes of the bacterial population to the presence of toxic substances in feeding, as well as to the combined toxicity exerted by biodegradation metabolites and parent compounds.

In conclusion, these results indicate that the biofilm is not severely affected by the presence of emerging contaminants in this pharmaceutical wastewater [50]. After an acclimatization period under extreme conditions, bacteria can form an electroactive biofilm capable of utilizing recalcitrant wastes as substrates [66], showing higher enzymatic activities as has been observed in other bioelectrochemical systems using electrodes [48].

It is interesting to remark that there is a trend in the chronoamperometric results along with the formation of ROS species. It has been reported that the exposure of bacterial biofilms to electrical current can cause partial death of cells [67], but as we previously show, some authors report that the electrical current is beneficial to stimulating the biological activity of the EAB [49].

Electroactive biofilms on graphite rod and carbon felt showed different acclimation periods to the pharmaceutical wastewater, achieving a constant electrical current after 9 and 15 days, respectively (Figure 3). However, after these periods, both systems show a clear correlation between electrical current and enzymatic activity and oxidative stress, i.e., ROS presence.

The COD removal yield is enhanced, increasing by 50% to 40% for felt and rod electrodes, respectively. This change is not reflected in the electrical current signal, but the most revealing information comes from the enzymatic activity analysis (Figure 1). After two weeks, the ROS species decreased dramatically, and the catalase concentration increased. This is the behavior observed in the enzymatic activities of planktonic cells (Figure 1b,c), whilst the biofilm probably exhibited the opposite behavior because the final enzymatic activities measured in biofilms were higher than in planktonic cells (Figure 1a). This phenomenon is not unexpected as long as the microorganisms mainly responsible for COD removal are the ones forming the EAB.

**Figure 4:**
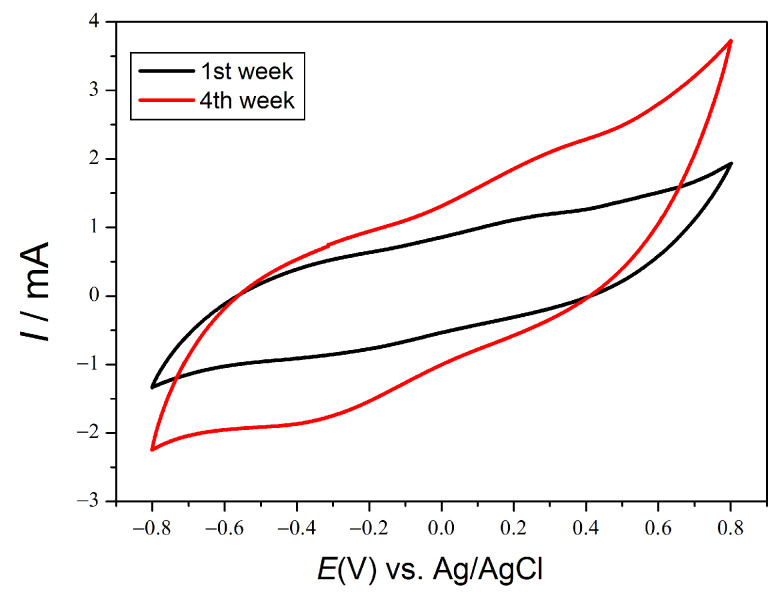
Cyclic voltammetry in rod anode.

In addition, when the electrochemical fingerprint of the EAB is observed through cyclic voltammetry essays, the change in the electrochemical response is clearly observed after two weeks, Figure 4. The electrical response of the EAB at positive potentials is significantly enhanced after two weeks compared to the early stages of acclimation.

These results showed for the very first time that the EAB was able to degrade complex organic pollutants, such as the ones present in pharmaceutical wastewater, through a combination of extracellular electron transfer and catalase production to control oxidative stress (ROS species).

## 4. Conclusions

The results obtained in this work demonstrated that EAB showed higher enzymatic activities than the planktonic cells in bioelectrochemical reactors. Catalase resulted similarly in cells forming biofilm in the MEC system, but esterase activity depended on the anodic architecture (carbon rod < carbon felt), also, esterase was higher in biofilm compared to planktonic cells. In contrast, ROS measured in free cells was higher than in the EAB attached to anodes, especially in the MEC-F system. This profile of enzymatic is a response to toxicant feeding showed that EAB developed an adaptative strategy, probably increasing catalase activity to counteract ROS generation caused by the toxic influent. This acclimatation permits EAB microorganisms to stimulate the mixed population for the removal and detoxification of pollutants.

Acclimatization resulted in a very difficult process for planktonic cells, which gradually lost their ability to counteract ROS (decreasing catalase activity along operation time) and lost cell integrity, as can be observed by the reduction in esterase activity.

COD removal and detoxification showed a similar trend, with a minimum of 15 days, reaching a stable value after that. In contrast, the current generation was almost stable and showed no major changes throughout the operation, so the EAB microorganisms were not severely affected.

The bacteria forming the electroactive biofilm, regardless of the anode architecture, tolerate the toxic impact of industrial effluent better than planktonic cells and maintain their biological activity by adapting to the presence of pollutants thanks to the generation of catalase, which enables oxidative stress to be controlled. However, the mechanisms controlling the oxidative stress response of electroactive bacterial populations need to be further studied to develop more effective treatment systems for industrial effluent treatment.

## Figures and Tables

**Table 1:** Experimental conditions and measured parameters.

Reactor	Electrode Materials		Process
Control	-	-	Anaerobic conventional
MEC-R	rod	felt	Microbial electrolysis cell
MEC-F	felt	felt	Microbial electrolysis cell

**Table 2:** Characteristics of real pharmaceutical wastewater used in this work.

Parameters	Mean Value with 95% Confidence Interval
COD (mg/L)	765 ± 30
pH	8.5 ± 0.2
Toxicity for *Vibrio fisheri* (Inhibition, %)	100 ± 1
Toxicity for *Spirodela polyrhiza* (Inhibition, %)	100 ± 1

## Data Availability

The original data presented in the study are included in the article; further inquiries can be directed to the corresponding author.
